# Vaccination with chemically attenuated *Plasmodium falciparum* asexual blood-stage parasites induces parasite-specific cellular immune responses in malaria-naïve volunteers: a pilot study

**DOI:** 10.1186/s12916-018-1173-9

**Published:** 2018-10-08

**Authors:** Danielle I. Stanisic, James Fink, Johanna Mayer, Sarah Coghill, Letitia Gore, Xue Q. Liu, Ibrahim El-Deeb, Ingrid B. Rodriguez, Jessica Powell, Nicole M. Willemsen, Sai Lata De, Mei-Fong Ho, Stephen L. Hoffman, John Gerrard, Michael F. Good

**Affiliations:** 1 Institute for Glycomics, Griffith University, Parklands Drive, Southport, Queensland Australia; 2 Gold Coast University Hospital, 1 Hospital Blvd, Southport, Queensland Australia; 3 Sanaria Inc., Gaithersburg, MD USA

**Keywords:** Malaria, *Plasmodium falciparum*, Vaccines, Chemically attenuated malaria parasites, T cell responses

## Abstract

**Background:**

The continuing morbidity and mortality associated with infection with malaria parasites highlights the urgent need for a vaccine. The efficacy of sub-unit vaccines tested in clinical trials in malaria-endemic areas has thus far been disappointing, sparking renewed interest in the whole parasite vaccine approach. We previously showed that a chemically attenuated whole parasite asexual blood-stage vaccine induced CD4^+^ T cell-dependent protection against challenge with homologous and heterologous parasites in rodent models of malaria.

**Methods:**

In this current study, we evaluated the immunogenicity and safety of chemically attenuated asexual blood-stage *Plasmodium falciparum* (Pf) parasites in eight malaria-naïve human volunteers. Study participants received a single dose of 3 × 10^7^ Pf pRBC that had been treated in vitro with the cyclopropylpyrolloindole analogue, tafuramycin-A.

**Results:**

We demonstrate that Pf asexual blood-stage parasites that are completely attenuated are immunogenic, safe and well tolerated in malaria-naïve volunteers. Following vaccination with a single dose, species and strain transcending *Plasmodium*-specific T cell responses were induced in recipients. This included induction of *Plasmodium*-specific lymphoproliferative responses, T cells secreting the parasiticidal cytokines, IFN-γ and TNF, and CD3^+^CD45RO^+^ memory T cells. Pf-specific IgG was not detected.

**Conclusions:**

This is the first clinical study evaluating a whole parasite blood-stage malaria vaccine. Following administration of a single dose of completely attenuated Pf asexual blood-stage parasites, *Plasmodium*-specific T cell responses were induced while Pf-specific antibodies were not detected. These results support further evaluation of this chemically attenuated vaccine in humans.

**Trial registration:**

Trial registration: ACTRN12614000228684. Registered 4 March 2014.

**Electronic supplementary material:**

The online version of this article (10.1186/s12916-018-1173-9) contains supplementary material, which is available to authorized users.

## Background

*Plasmodium* spp. parasites cause more than 200 million clinical cases of malaria and 438,000 deaths per year, with the majority of deaths occurring in children < 5 years of age [1]. An effective vaccine capable of inducing long-lasting immunity is not currently available. Disappointing results following the testing of sub-unit vaccines in clinical trials [2–5] have highlighted some of the limitations of sub-unit vaccines that need to be addressed, including antigenic polymorphism in critical epitopes.

The limited protection induced by sub-unit vaccine candidates has resulted in renewed interest in the whole organism vaccine approach. The fundamental rationale for a whole parasite vaccine is that by maximising the number of antigens presented to the immune system, including those that are conserved between different parasite strains, the impact of antigenic polymorphism will be diminished. There has been considerable progress with injectable whole parasite *Plasmodium falciparum* sporozoite (PfSPZ) vaccines [6–11]. The administration of whole blood-stage parasites in the context of controlled human malaria infection (CHMI) in human volunteers is not new [12]; deliberate malaria infection was used as a treatment for neurosyphilis (malariotherapy) in the early 1900s (reviewed in [13, 14]). CHMI with whole blood-stage parasites is also used for the in vivo assessment of malaria vaccine and drug candidate efficacy (reviewed in [12]). However, there have been no published clinical studies of whole parasite blood-stage malaria vaccines [15].

Cyclopropylpyrolloindole analogues, such as centanamycin (CM) and tafuramycin-A (TF-A) have been used to successfully attenuate both sporozoite and asexual blood-stage malaria parasites [16–20]. These compounds bind covalently to poly-A regions of DNA [21]. Studies in mice involving vaccination with chemically attenuated sporozoites demonstrated induction of protective immunity [16, 17]. To adapt this for a blood-stage vaccine approach, we vaccinated mice with a single dose of ring-stage *Plasmodium chabaudi* AS parasitised red blood cells (pRBC) that had been treated in vitro with CM or the related compound, TF-A, and demonstrated long-lasting protection from homologous and heterologous blood-stage challenge [18]. Similar protection was observed when mice were vaccinated with chemically attenuated *Plasmodium yoelii* 17X, although three doses of vaccine provided superior protection compared to one dose [19]. Although an adjuvant was not required for induction of protective immune responses, vaccine efficacy was ablated if the red cell membrane was disrupted [18]. These data suggested that the red cell membranes were required to target the attenuated parasites to dendritic cells in the spleen and liver, which was observed post-vaccination. Protective immunity was dependent on CD4^+^ T cells present at the time of challenge, and a strong IFN-γ response was induced by the vaccine [18, 19]. Parasite-specific antibodies were induced only in the *P. yoelii* 17X model and contributed to protection. Vaccination also led to a significant CD8^+^ T cell response, although depletion of these cells did not ablate vaccine-induced immunity. In previous pre-clinical studies involving other types of whole parasite blood-stage vaccines, it was shown that cellular immunity or IFN-γ played critical roles in protection [22–24]. The importance of IFN-γ in immunity to human malaria has also been demonstrated in individuals in malaria-endemic areas [25–28] and in a controlled human experimental infection study [29].

To facilitate transition of the chemically attenuated vaccine approach into humans, pre-clinical in vitro and in vivo studies with *P. falciparum* (Pf) were undertaken. Treatment of parasites with 2 μM CM resulted in complete parasite attenuation in vitro [18]. In vivo studies in *Aotus* monkeys showed that following inoculation of TF-A-treated parasites, they persisted at sub-patent levels for up to 8 days (as determined by qPCR) [30]. Pf-specific T cell responses, but not Pf-specific IgG, were induced. Collectively, these data supported the evaluation of this vaccine approach in clinical studies.

We previously manufactured clinical-grade cultured Pf asexual blood-stage cell banks [31] and demonstrated their infectivity in vivo in malaria-naïve volunteers [32]. In this present study, we used the Pf 7G8 cell bank to investigate the immunogenicity, safety and tolerability of chemically attenuated parasites in malaria-naïve individuals.

## Methods

### Aims and study participants

The main aims of the study were to (i) characterise the safety and tolerability of TF-A-treated Pf blood-stage parasites in humans and to (ii) characterise the immunogenicity of TF-A-treated Pf blood-stage parasites in humans. Griffith University was the study sponsor, and the study was conducted in the Clinical Trial Unit at Griffith University, Southport, Queensland, Australia, from July 2014 to August 2015. Study participants were healthy male, malaria-naïve individuals, aged 18–60 years (*n* = 8) (Additional file 1: Table S1). Volunteers were excluded if they had a history of malaria infection or travelled to/lived (> 2 weeks) in a malaria-endemic country during the previous 12 months. Other key eligibility criteria can be found in the listing on the Australian New Zealand Clinical Trials registry (www.anzctr.org.au); the identifier is ACTRN12614000228684.

Study participants received a single vaccination of 3 × 10^7^pRBC treated with 50 nM of TF-A (group A; *n* = 3) or 200 nM of TF-A (group B; *n* = 5) on study day 0. Follow-up visits were scheduled every 2 days (from study day 2 to day 26) following vaccination. At these visits, blood samples were collected to assess parasite levels in the blood of participants and to evaluate the immunogenicity of the vaccine in established assays. If the numbers of parasites in the blood increased exponentially and levels reached 11,500 pRBC/ml (as measured by quantitative PCR [qPCR]) or clinical symptoms of malaria developed, rescue treatment with a standard course of the anti-malarial artemether-lumefantrine (A/L) (Riamet) was commenced immediately. If rescue treatment with A/L was not initiated, 4 weeks following administration of the vaccine (day 28), participants were given a standard course of A/L.

For safety assessments at every visit, participants were evaluated by a medical investigator. This included a physical exam, measurement of vital signs (e.g. temperature, heart rate, blood pressure and respiratory rate) and recording of solicited and unsolicited adverse events. Blood was also collected for safety purposes at designated scheduled visits (days 0, 8, 16, 28, 90) for group B. For group A, this was undertaken at days 0, 8, the day of initiation of anti-malarial treatment for each participant (days 10–13), days 28 and 90. Sullivan Nicolaides Pathology tested samples collected prior to immunisation, on day 28 and day 90 for the presence of alloantibodies. Indirect anti-globulin testing was undertaken using column agglutination technology. An independent Safety Review Team, including an independent medical expert, was appointed to oversee the study and monitor its progress.

### Culture of Pf for the production of chemically treated parasites

The culturing of Pf 7G8 for the production of chemically treated parasites was undertaken at Griffith University. All processes were carried out in compliance with Annex 13, Pharmaceutical Inspection Co-operation Scheme (PIC/S) Guide, in a monitored environment suitable for production of sterile biologics in accordance with approved protocols. For group A (P1, P2, P3) and three participants in group B (P4, P5, P6), cultures were initiated using seed vials from the clinical-grade Pf 7G8 cell bank [31] and were expanded using leukocyte-depleted group O RhD negative erythrocytes (Key Biologics, LLC, Memphis, TN, USA) as previously described for the production of the clinical-grade cell banks [31]. For two participants in group B (P7 and P8), the Pf 7G8 cell bank was expanded in erythrocytes derived from the blood of the study participant. Parasite cultures were checked regularly, at which time, thin blood films were made from collected samples, stained with Diff Quik (Bacto Laboratories) and read to ascertain the parasitemia. As required, the parasites were sub-cultured using freshly washed human erythrocytes. This culturing process was continued with the number of tissue culture dishes/flasks increasing until the malaria parasite was at ring-stage, and it was calculated that there were sufficient parasite numbers to manufacture the chemically treated pRBC.

### Chemical treatment of Pf 7G8 with tafuramycin-A

The 2 mM TF-A stock solution was prepared according to published methods [18], and aliquots were stored at − 80 °C. Fresh working stocks of 20 μM were made from this as required, and serial dilutions were performed in Roswell Park Memorial Institute (RPMI)-1640 (Gibco, Invitrogen Corporation, CA) to obtain the appropriate concentration of TF-A for the chemical treatment of pRBCs. Pf 7G8 cultures were centrifuged at 433*g* for 10 min, and the supernatant removed. The cell pellets were combined in a single tube, and a thin blood smear was prepared to determine parasitemia. The parasitemia of cultures for preparation of chemically treated pRBC was 3–5%. For each vented flask required, 500 μl of packed cells (pRBCs and uninfected red blood cells [uRBCs]) was added to 9 ml of pre-warmed RPMI-1640 medium. One millilitre of the appropriate TF-A solution was added to obtain a final concentration of either 50 nM (group A) or 200 nM of TF-A (group B). This cell suspension was incubated for 40 min in a 37 °C incubator with 5% O_2_, 5% CO_2_, and 90% N_2_, and the flasks were gently agitated every 10 min. The packed cells were transferred to 50 ml conical tubes and washed with RPMI-1640 at 433*g* for 5 min, and the supernatant discarded. The pellet was resuspended in RPMI-1640 and incubated at 37 °C for a further 20 min. The pRBC were washed twice more with RPMI-1640 and a final wash in 0.9% saline for injection. Finally, the pellet was re-suspended in saline for injection and a cell count was performed to calculate the volume required for the immunising dose. This was re-suspended in saline for injection to give a final volume of 2 ml/dose.

### Preparation and administration of the chemically treated Pf vaccine

The vaccine was dispensed into as many 2 ml syringes as required for administration to the study participants who were inoculated by intravenous injection. Study participants received an inoculum containing either 3 × 10^7^ Pf 7G8 pRBC treated with 50 nM of TF-A (group A) or 3 × 10^7^ Pf 7G8 pRBC treated with 200 nM of TF-A (group B). The number of parasites present in each batch of vaccine was verified retrospectively by undertaking qPCR on surplus material.

### Evaluation of the chemically treated Pf vaccine

During preparation of each batch of chemically attenuated inocula, additional inocula were prepared in parallel for testing as described below.

### Sterility testing of the chemically treated Pf vaccine

Sterility testing of in-process samples and inocula for the assessment of biocontamination with aerobic and anaerobic microorganisms was undertaken by Biotest Laboratories Pty Ltd. (Underwood, Australia) using the direct inoculation technique into tryptone soya broth and thioglycollate medium. Test parameters and acceptance criteria were defined according to the British Pharmacopoeia 2014, Appendix XVI A. Following a 14-day incubation period, there was no evidence of growth of aerobic or anaerobic microorganisms.

### Measurement of residual tafuramycin-A in chemically treated vaccine

A bioanalytical method to determine the residual TF-A in a vaccine dose was developed and qualified by the Centre for Integrated Preclinical Drug Development (CIPDD), Herston, Australia). The range of detection of the assay was 5–200 ng/ml. A vaccine dose from each batch was frozen on dry ice and sent to CIPDD for analysis. During the manufacturing process, the majority of the TF-A is washed away; any residual compound is considered a by-product of manufacture and an impurity in the final product. In all batches produced, the amount of residual TF-A present was well below the limit described in the “European Union (EU) Guidelines on the limits of Genotoxic Impurities” of 1.5 μg/person/day (group A: *x* = 86.04 ng/vaccine dose; range: 14.4–206.8 ng/vaccine dose; and group B: *x* = 114 ng/vaccine dose; range:82.4–136.8 ng/vaccine dose).

### Growth of parasites, as assessed by tritiated hypoxanthine uptake

The viability of the parasites following chemical attenuation was assessed using the [^3^H]-hypoxanthine growth inhibition assay. Chemically attenuated ring-stage parasites (2% haematocrit) were added to 96-well flat-bottomed plates (100 μl per well) in quadruplicate. Unattenuated ring-stage parasites and unparasitised red blood cells (uRBC) at 2% haematocrit were used as positive and background controls respectively. Plates were placed in a 37 °C incubator with 5% O_2_, 5% CO_2_, and 90% N_2_. The assay duration was 48 h with [^3^H]-hypoxanthine (0.2 μCi/well) added from the start of the experiment. Following incubation, plates were frozen, then subsequently thawed and harvested onto glass fibre mats (Perkin Elmer, Australia) using a Filtermate cell harvester (Perkin Elmer). Radioactivity was measured using a Microbeta^2^ counter (Perkin Elmer). The remainder of the packed cells from the vaccine were placed in culture, and after 1 week, 2 weeks and 3 weeks of culture, cells were harvested and evaluated according to incorporation of [^3^H]-hypoxanthine. Twice a week, fresh uRBC were added into the cultures and the medium changed. No growth was observed, as measured by lack of [^3^H]-hypoxanthine incorporation, compared to unattenuated Pf 7G8 control samples that were cultured in parallel.

### PCR

Sample preparation, DNA extraction and parasitemia, as measured by qPCR, were undertaken as previously described [33] with the following modifications. The standard curve was prepared from a lyophilized World Health Organisation (WHO) Pf international standard (NIBSC code: 04/176) [34] that was reconstituted in 500 μl of nuclease-free water and diluted in a 1:1 solution with 1 X phosphate buffered saline (PBS) (Gibco). DNA was isolated from 500 μl of this solution at a concentration of 5 × 10^8^ IU/ml. Blood samples from study participants and standards were tested in triplicate. Established modified calculations [35] were used to equate international units (IU)/ml to parasites/ml, with 1 IU/ml equivalent to 0.5 parasites/ml. The number of parasites/ml was calculated using the CFX96 Touch Real Time Detection System software (BioRad, Australia).

### Collection and processing of samples from study participants

Whole blood was collected from study participants in sodium heparin tubes and centrifuged at 433*g* for 10 min. Plasma was removed and stored at − 80 °C until it was required for analysis. The cell pellet was diluted 1:1 in RPMI-1640, and peripheral blood mononuclear cells (PBMCs) were isolated by density centrifugation with Ficoll-Paque (Amersham). PBMCs were washed, resuspended at 1 × 10^7^ cells/ml in 90% heat inactivated foetal bovine serum (FBS)/10% dimethyl sulfoxide and frozen to − 80 °C at 1 °C/min in freezing containers for 24 h (Nalgene), before transfer to liquid N_2_ for storage.

### Enzyme-linked immunosorbent assay (ELISA)

NUNC Maxi-sorp immunoplates (Thermoscientific, Australia) were coated with 5 μg/ml of crude Pf 7G8 antigen in bicarbonate coating buffer, pH 9.6 and incubated overnight at 4 °C. After washing with 0.05% Tween20/PBS, plates were blocked with 10% skim milk buffer/0.05% Tween 20/PBS and incubated at 37 °C for 2 h. Following washing, plasma (diluted 1:50 in 5% skim milk buffer/0.05% Tween 20/PBS) was added to the plates and they were incubated at 37 °C for 1 h. The plates were washed again, and a goat anti-human IgG horseradish peroxidase conjugate (Abcam, Australia) or a goat anti-human IgM Fc5μ horseradish peroxidase conjugate (Merck Millipore) was added at 1:10,000 (IgG) or 1:2,500 (IgM) in 5% skim milk buffer/0.05% Tween 20/PBS and plates were incubated at 37 °C for 1 h. Following further washing, tetramethylbenzadine (TMB) substrate solution (Becton Dickinson, Australia) was added and plates were incubated at room temperature for 10–15 min. Absorbance was measured at 650 nM on an xMark micro-plate reader (Bio-rad, Australia). Positive control serum was obtained from residents of malaria-endemic areas. Negative control serum was obtained from unexposed Brisbane residents.

### PBMC stimulation assays

Upon thawing, cells were washed thrice in complete medium (RPMI-1640 containing 10% heat inactivated human serum, 2 nM L-glutamine, 100 U/ml of penicillin and 100 mg/ml of streptomycin sulphate), re-suspended in complete medium, counted using trypan blue (Sigma) and aliquoted into U-bottom 96-well plates.

For T cell proliferation assays, 2 × 10^5^ cells in 100 μl was added per well. Subsequently, 100 μl of purified fresh pRBCs at trophozoite/schizont stage (Pf 7G8, Pf NF54 or *Plasmodium knowlesi* A1H1.1) or uRBCs (6 × 10^5^ cells/well), 1% phytohaemagglutinin (PHA; Gibco) or media only was added, and PBMCs were cultured for 7 days at 37 °C, 5% CO_2_. Each treatment was tested in triplicate.

For intracellular cytokine staining, 5 × 10^5^ cells in 100 μl were added per well. Subsequently, 100 μl of purified fresh Pf 7G8 pRBCs or uRBCs (1 × 10^6^ cells/well), 1% PHA or media only was added, and PBMCs were cultured for 36 h at 37 °C, 5% CO_2_. Each treatment was tested in triplicate. Sorbitol-synchronised, *Mycoplasma*-negative, live, late-stage trophozoite/schizont stage pRBCs used in the above in vitro assays were purified by magnetic separation over CS columns (Miltenyi Biotec) on a VarioMACs magnet (Miltenyi Biotec) for these assays.

### Measurement of PBMC proliferation

For assessing proliferation of PBMCs via the incorporation of radioisotope, the unlabelled cells were pulsed with 1 μCi of ^3^[H]-thymidine/well (Perkin Elmer, Australia) for the final 18 h and plates were stored at − 80 °C. Following thawing, cells were harvested onto glass fibre mats (Perkin Elmer, Australia) using a Filtermate cell harvester (Perkin Elmer) and radioactivity was measured using a β-scintillation microplate counter (Perkin Elmer). The uptake of ^3^[H]-thymidine was measured as corrected counts per minute (CCPM), and results were expressed as deltaCPM, which is defined as the ^3^[H]-thymidine (CPM) in the presence of stimulus, subtracting the average ^3^[H]-thymidine (CPM) incorporated in the presence of the appropriate control stimulus (e.g. unparasitised red blood cells).

### Detection of cytokines by cytometric bead array

After 6 days of culture, prior to the addition of radioisotope, cell culture supernatants were removed and frozen at − 80 °C. Cytokines were measured in the thawed culture supernatants using a Th1/Th2/Th17 cytometric bead array (CBA) kit (BD Biosciences) according to the manufacturer’s instructions. Samples were analysed on a CyAn ADP flow-cytometer, and data analysis was performed using BD FCAPArray software. To determine agonist-specific cytokine induction, background levels from uRBC alone were subtracted. Selected plasma samples were also analysed with Th1/Th2/Th17 CBA kits according to the manufacturer’s instructions.

### Identification of cellular sources of cytokines by flow cytometry

For the final 4 h of incubation, Golgi-Plug (BD Biosciences) was added. Plates were removed from the incubator and centrifuged at 433*g* for 5 min. To enable dead cell exclusion, the LIVE/DEAD Aqua Fixable Dead Cell Stain (Thermofisher Scientific) was added to the cells according to manufacturer’s instructions and incubated in the dark at room temperature for 30 min. Following washing, antibodies for staining of cell surface markers (γδ TCR PE-CF594, clone B1; CD3 PerCp, clone SK7; CD4 450, clone RPA-T4; CD8 PECy7, clone RPA-T8; CD45RO APC-H7, clone UCHL1; all from BD Biosciences) were diluted in FACs buffer (1% bovine serum albumin [BSA]/PBS), added to cells and incubated for 20 min in the dark on ice. Following washing with FACs buffer, cells were fixed in 40% *v*/*v* formalin at room temperature for 15 min. Cells were fixed and permeabilised using the BD Fix/Perm Kit (BD Biosciences) according to the manufacturer’s instructions. Intracellular staining with cytokine-specific antibodies (IFN-γ APC, clone B27; TNF FITC, clone 6401.1111; IL-2 PE, clone MQ1-17H12; all from BD Biosciences) and the appropriate isotype controls was performed on ice for 30 min. Following washing, cells were resuspended in FACs buffer for analysis of the Cyan ADP flow cytometer (Beckman Coulter, Australia). Data analysis was performed using FlowJo V10 (FlowJo, LLC).

### Statistics

All data were analysed and graphed using GraphPad PRISM 6. One-way ANOVA was performed on datasets followed by Dunnett’s multiple comparisons test. For the antibody and T cell proliferative data, analyses were conducted at an individual level, using assay replicates and comparing responsiveness at day 0 with subsequent time points. For all other immunological analyses, data was combined for all individuals within a group at each time point, and comparisons were conducted between day 0 and subsequent time points.

## Results

### Parasite growth in volunteers post-inoculation

We initially established the minimal dose of TF-A necessary to completely attenuate Pf 7G8 and prevent growth of the parasite in vitro. We observed that a dose of 50 nM was sufficient as demonstrated by the lack of parasite growth measured by ^3^[H] hypoxanthine incorporation (Additional file 1: Figure S1). We then produced vaccine doses for administration to volunteers. The biological properties of the vaccine, including assessment of residual TF-A are described in the “Methods” section.

**Fig. 1:**
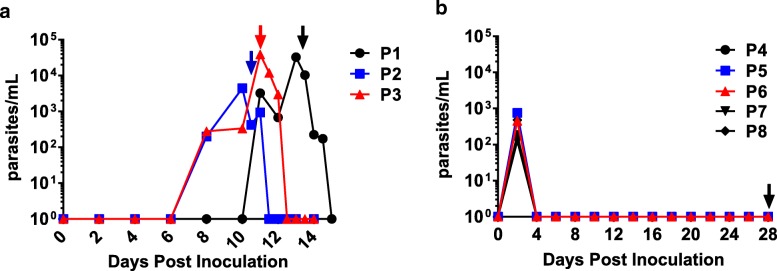
The course of parasitemia in study participants inoculated with chemically treated *P. falciparum* 7G8. Parasite levels in study participants, as determined by qPCR, following inoculation with **a** 3 × 10^7^*P. falciparum* pRBC treated with 50 nM tafuramycin-A (TF-A) or **b** 3 × 10^7^*P. falciparum* pRBC treated with 200 nM TF-A. Arrows indicate initiation of drug treatment with artemether-lumefantrine

We treated three study participants in group A (P1 → P3), with a single vaccine dose containing 3 × 10^7^ pRBC treated with 50 nM TF-A. The dose of 3 × 10^7^ pRBC was chosen based on the lowest dose of a *P. chabaudi*-attenuated vaccine shown to be efficacious in mice (10^4^) [18], with a correction for approximate weight differences. Surprisingly, all three participants developed a sub-patent Pf infection (Fig. 1a) necessitating initiation of anti-malarial treatment with A/L on days 10–13 (according to symptoms and the parasitemia threshold as defined in the study protocol [11,500 parasites per millilitre]). As a TF-A concentration of 50 nM was insufficient to completely attenuate the parasite, a higher dose was used to prepare the vaccine for the next study group (group B), which received a single vaccine dose of 3 × 10^7^ pRBC treated with 200 nM TF-A. Apart from a sub-patent parasitemia detected by qPCR on day 2 only, all five participants (P4 → P8) remained parasite-negative until day 28, when drug treatment with A/L was initiated in accordance with the study protocol (Fig. 1b).

### Adverse events and lab abnormalities

A number of adverse events (AEs) and abnormal laboratory values considered probably or possibly related to the vaccine were recorded for participants in group A (Additional file 1: Tables S2 and S3). The majority of these were typical of symptoms or blood abnormalities observed during Pf infection (malaria) and resolved following completion of anti-malarial drug treatment. There were no AEs attributable to the vaccine recorded for the participants in group B.

### Induction of alloantibodies

Blood group O RhD negative blood was used to manufacture the chemically attenuated pRBC inoculum for P1 → P6. However, we observed that P6 seroconverted to the minor Rh antigen “c” by day 28. While his Rh phenotype was “CDe”, and the phenotype of the donor red blood cells was “ce”, this was an unexpected finding as it had not been observed in any of > 380 volunteers previously given controlled human blood-stage malaria infections (J McCarthy, pers. comm. and DI Stanisic, unpublished data). As a result of this finding, the inocula for the last two volunteers in group B (P7 and P8) were manufactured using their own blood.

### Induction of parasite-specific antibody responses

**Fig. 2:**
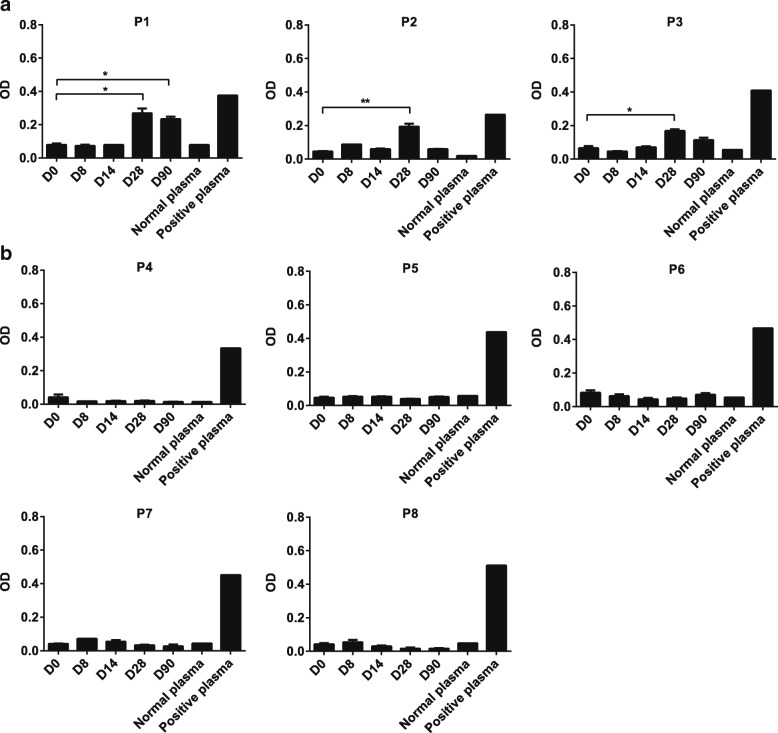
Induction of *P. falciparum* 7G8-specific IgM responses in study participants inoculated with **a** 3 × 10^7^*P. falciparum* 7G8 pRBC treated with 50 nM TF-A or **b** 3 × 10^7^*P. falciparum* 7G8 pRBC treated with 200 nM TF-A. ELISAs were performed to detect IgM specific for crude *P. falciparum* 7G8 antigen using plasma collected at different time points following vaccination. Results are expressed as optical density (OD) at 650 nm. Samples were run in duplicate. Data represents mean ± SEM. An individual’s data was analysed using a one-way ANOVA followed by Dunnett’s multiple comparisons test; *p < 0.01, **p < 0.001

Plasma samples from study participants were tested for Pf 7G8-specific IgM and IgG by ELISA. In all participants in group A, the group that developed Pf infection, parasite-specific IgM was induced, with significantly higher levels present on day 28 compared to day 0 (*p* < 0.01 for P1 and P3; *p* < 0.001 for P2) (Fig. 2a). Levels of parasite-specific IgM in group B (Fig. 2b), the group that did not develop Pf infection, and IgG in groups A (Additional file 1: Figure S2A) and B (Additional file 1: Figure S2B) were not significantly elevated compared to day 0 during the course of the study (*p* > 0.05).

### Induction of parasite-specific cellular responses

**Fig. 3:**
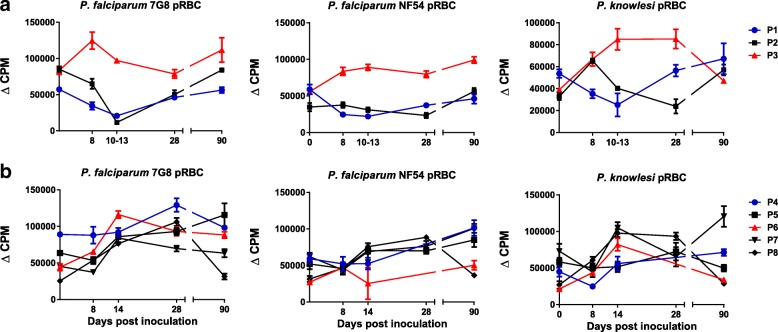
Lymphoproliferative responses to homologous (*P. falciparum* 7G8) and heterologous (*P. falciparum* NF54 and *P. knowlesi*) pRBC in study participants inoculated with a single dose of **a** 3 × 10^7^*P. falciparum* 7G8 pRBC treated with 50 nM (group A) or **b** 200 nM (group B) TF-A. Peripheral blood mononuclear cells (PBMCs) were isolated from blood samples collected at different time points post-inoculation and cryopreserved. Following thawing, PBMCs were incubated with parasitised red blood cells (pRBC) or unparasitised red blood cells (uRBC) for 7 days; the last 18 h with ^3^[H] thymidine. Proliferation of PBMCs was estimated by ^3^[H] thymidine incorporation. Data represents mean ± SEM for each time point (tested in triplicate). CPM: counts per minutes. Delta CPM indicates that responses to pRBC were corrected against responses to uRBC. The day 28 samples for P4 and P6 were not available for testing against *P. falciparum* NF54 and *P. knowlesi*

To assess cellular responses, parasite-specific lymphoproliferation (as measured by ^3^[H]-thymidine incorporation) was assessed to homologous (7G8) and heterologous (PfNF54 and *P. knowlesi*) pRBC. In group A, responses to homologous parasites (7G8) did not increase significantly compared to day 0 (*p* > 0.05) (Fig. 3a). There was a decrease in responses between days 8–13, which was associated with the development of infection and the administration of anti-malarial treatment (Fig. 1). Proliferative responses to the heterologous parasites did not increase at any time point for P1 (*p* > 0.05) (Fig. 3a). For P2 and P3, significantly increased responses compared to day 0 were seen to PfNF54 and *P. knowlesi* at various time points (*p* < 0.05) (Fig. 3a).

In group B, proliferative responses to homologous parasites (Fig. 3b) were significantly increased at one or more time points compared to day 0 for all five individuals (*p* < 0.04 for all); for 3/5 individuals, this was observed at three or more time points. Additionally, for 3/5 individuals, this was observed at D90. For heterologous parasites, significantly increased responses were seen to PfNF54 at one of more time points in all participants (*p* ≤ 0.02 for all) and to *P. knowlesi* in 4/5 study participants (P4, P6, P7 and P8) (*p* < 0.05 for all) (Fig. 3b).

**Fig. 4:**
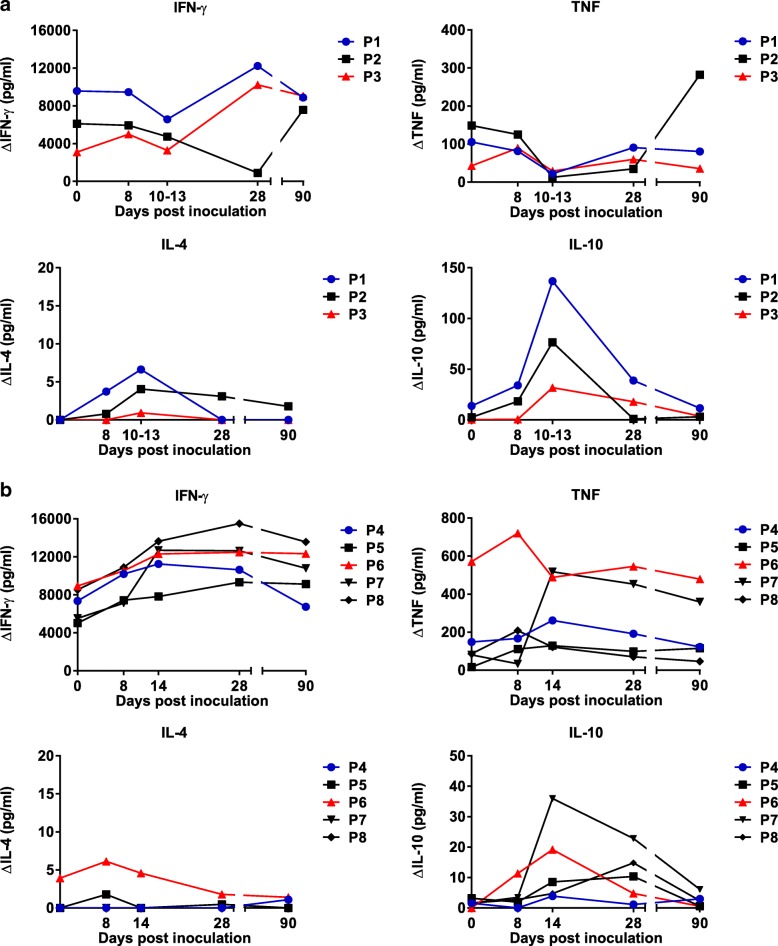
Cytokine responses to *P. falciparum* 7G8 in study participants inoculated with a single dose of **a** 3 × 10^7^*P. falciparum* 7G8 pRBC treated with 50 nM (group A) or **b** 200 nM (group B) TF-A. Peripheral blood mononuclear cells (PBMCs) were isolated from blood samples collected at different time points post-inoculation and cryopreserved. Following thawing, PBMCs were incubated with parasitised red blood cells (pRBC) or unparasitised red blood cells (uRBC) for 7 days. Eighteen hours before the end of the culture period, culture supernatants were collected, pooled (*n* = 3) and used in cytokine bead arrays to quantify the level of cytokines produced in response to *P. falciparum* 7G8 pRBCs by flow cytometric analysis. Delta cytokine indicates that responses to pRBC were corrected against responses to uRBC

Cytokines present in the supernatants of the PBMC cultures in the 7-day assay following incubation with *P. falciparum* 7G8 pRBC were measured. Similar to the lymphoproliferative responses, for group A, the production of IFN-γ, TNF and IL-6 generally decreased compared to day 0 between days 8–13; in most instances, this returned to baseline levels by D90 (Fig. 4a and Additional file 1: Figure S3A). IL-4 and IL-10 levels increased in all individuals in parallel with the decrease in inflammatory cytokines. When combining data for all individuals in group A at each time point, there was a significant increase in IL-10 production at day 14 (*p* = 0.018) compared to day 0. Production of IL-2 and IL-17A was not consistent between individuals (Additional file 1: Figure S3A). In group B, an increase in IFN-γ, TNF and IL-10 production compared to day 0 was observed for all individuals (Fig. 4b). When combining data for all individuals in group B at each time point, for IFN-γ, this increase was significant at days 14 and 28 (*p* < 0.02 for both), and for IL-10, it was significant at day 14 (*p* = 0.043). Production of IL-2, IL-4, IL-6 and IL-17A varied over time and between individuals (Fig. 4b and Additional file 1: Figure S3B).

We were interested in the endurance of the altered immune response following vaccination. Four of the five individuals in group B demonstrated persisting altered immune responses, compared to day 0, to *P. falciparum* 7G8 pRBC resulting in production of the parasiticidal cytokine, IFN-γ, at day 90 and two of these individuals also had persisting TNF responses (Fig. 4b). The one individual (P4) whose response did not persist to day 90 was responsive until day 28.

Due to the appearance of clinical symptoms in participants in group A, we evaluated plasma levels of key inflammatory and anti-inflammatory cytokines in these individuals and compared these levels with those in plasma from participants in a previous study where we had evaluated the infectivity of the Pf 7G8 cell bank [32]. Similar parasitemias were observed in those study participants [32], who were asymptomatic at the time of initiation of A/L treatment and in whom it was initiated according to the same criteria for reaching the parasitemia threshold. Overall, higher levels of IL-6 and IL-10 were observed in individuals in the current study compared with the previous infectivity study (Additional file 1: Figure S4).

**Fig. 5:**
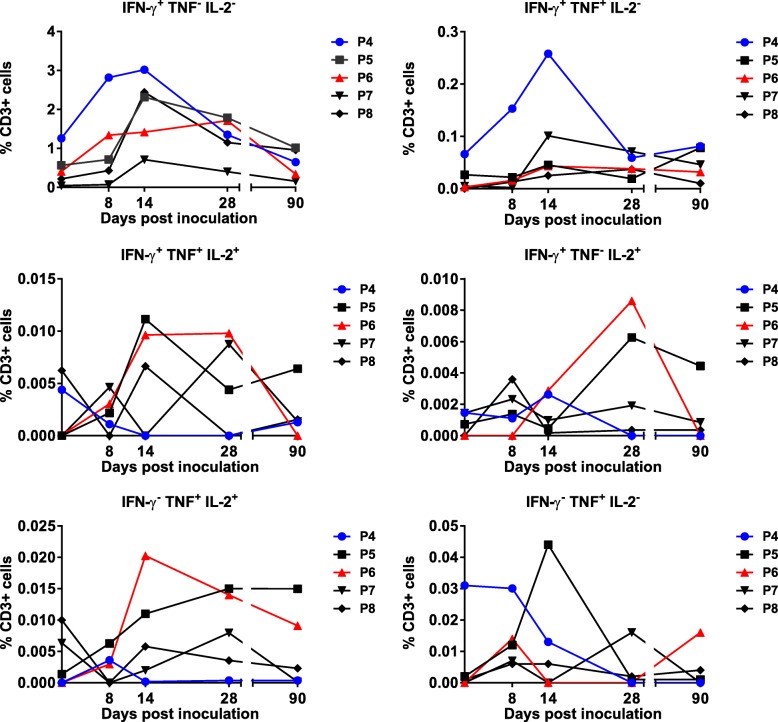
Monofunctional and polyfunctional CD3^+^ T cells in study participants inoculated with a single dose of 3 × 10^7^*P. falciparum* 7G8 pRBC treated with 200 nM TF-A (group B). Peripheral blood mononuclear cells (PBMCs) were isolated from blood samples collected at different time points post-inoculation and cryopreserved. Following thawing, PBMCs were incubated with parasitised red blood cells (pRBC) or unparasitised red blood cells (uRBC) for 36 h. Cells from triplicate wells were collected and pooled prior to staining with antibodies for flow cytometric analysis to evaluate the proportion of CD3^+^ T cells producing intracellular IFN-γ, TNF and IL-2. Responses to pRBC were corrected against responses to uRBC

The intracellular production of IFN-γ, TNF and IL-2 in response to homologous pRBC in short-term in vitro assays was also examined in CD3^+^ T cells. Initially, we examined CD3^+^ T cells to identify monofunctional and polyfunctional T cells secreting the parasiticidal cytokines, IFN-γ and TNF. T cells secreting these cytokines individually or in combination were induced in both groups following inoculation (Fig. 5 and Additional file 1: Figure S5). In group B, T cells secreting IFN-γ alone or in combination with TNF were the most frequently detected (Fig. 5). When combining data for all individuals in group B at each time point, there was a significant increase in cells secreting IFN-γ alone (i.e. IFN-γ^+^TNF^−^IL-2^−^) when comparing day 14 with day 0 (*p* < 0.02). Triple cytokine-secreting cells (IFN-γ, TNF and IL-2) were also detected, albeit at lower frequencies.

**Fig. 6:**
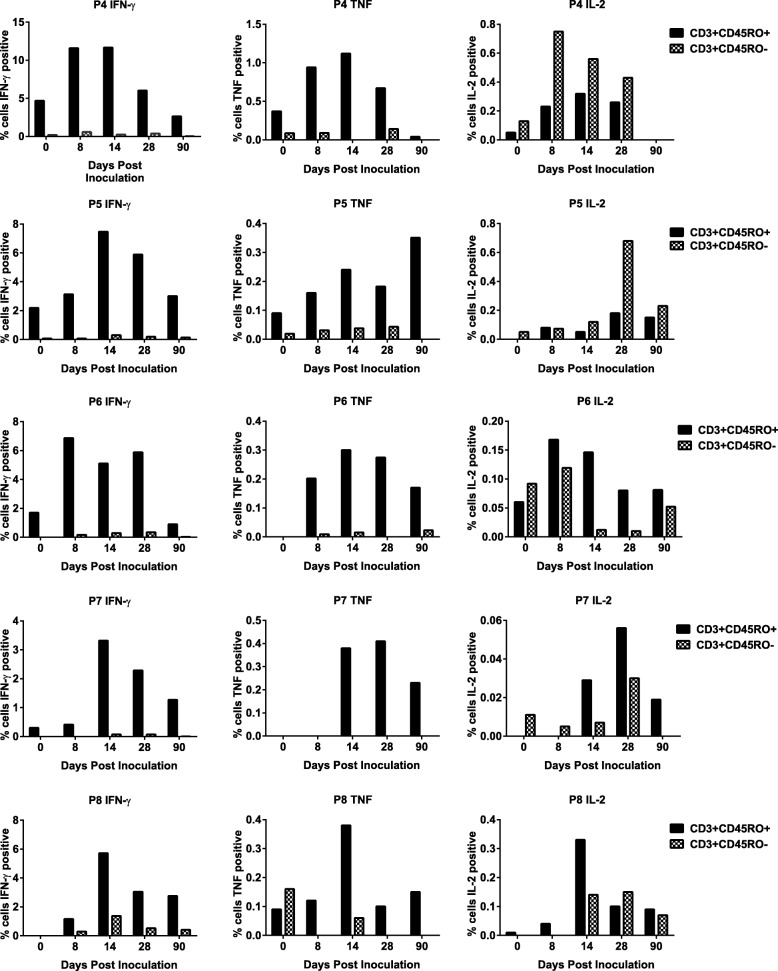
Cytokine production in naïve and memory T lymphocytes in study participants inoculated with a single dose of 3 × 10^7^*P. falciparum* 7G8 pRBC treated with 200 nM TF-A (group B). Peripheral blood mononuclear cells (PBMCs) were isolated from blood samples collected at different time points post-inoculation and cryopreserved. Following thawing, PBMCs were incubated with parasitised red blood cells (pRBC) or unparasitised red blood cells (uRBC) for 36 h. Cells from triplicate wells were collected and pooled prior to staining with antibodies for flow cytometric analysis to evaluate the proportion of naïve T cells (CD3^+^CD45RO^−^) and memory T cells (CD3^+^CD45RO^+^) producing intracellular IFN-γ, TNF and IL-2. Responses to pRBC were corrected against responses to uRBC

Intracellular production of the three cytokines in response to homologous pRBC was then examined individually in naïve (CD3^+^CD45RO^−^) and memory (CD3^+^CD45RO^+^) T cell populations. In both groups, all three cytokines were produced by both cell types with the cellular source and cytokine profile varying between individuals (Fig. 6 and Additional file 1: Figure S6). Importantly, memory T cells (CD3^+^CD45RO^+^) producing the three cytokines were induced following inoculation in all individuals in group B following inoculation (Fig. 6). When combining data for all individuals in group B at each time point, CD3^+^CD45RO^+^ cells secreting IFN-γ were significantly increased at day 14 compared with day 0 (*p* = 0.04).

**Fig. 7:**
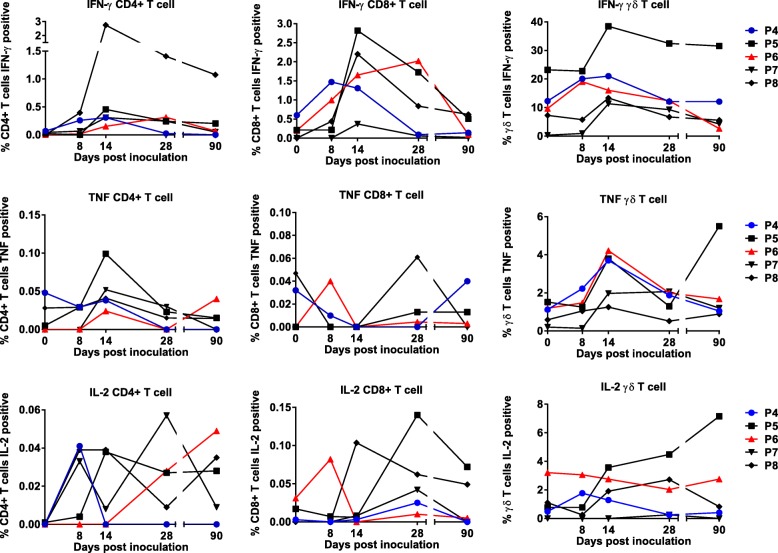
Cytokine production in CD3^+^ lymphocyte sub-populations in study participants inoculated with a single dose of 3 × 10^7^*P. falciparum* 7G8 pRBC treated with 200 nM TF-A (group B). Peripheral blood mononuclear cells (PBMCs) were isolated from blood samples collected at different time points post-inoculation and cryopreserved. Following thawing, PBMCs were incubated with parasitised red blood cells (pRBC) or unparasitised red blood cells (uRBC) for 36 h. Cells from triplicate wells were collected and pooled prior to staining with antibodies for flow cytometric analysis to evaluate intracellular IFN-γ, TNF and IL-2 production in helper T cells (CD3^+^CD4^+^CD8^−^), cytotoxic T cells (CD3^+^CD4^−^CD8^+^) and γδ T cells (CD3^+^ γδ^+^). Responses to pRBC were corrected against responses to uRBC

Intracellular cytokine production was also examined according to CD3^+^ T cell subset: helper T cells (CD3^+^CD4^+^CD8^−^); cytotoxic T cells (CD3^+^CD4^−^CD8^+^); and γδ T cell (CD3^+^γδ^+^) populations. There was heterogeneity in the cellular source and cytokine profile in individuals across both groups A and B (Fig. 7 and Additional file 1: Figure S7). Generally, in group B, with the exception of TNF production in CD8^+^ T cells, IFN-γ and TNF production by the different T cell subsets increased following inoculation and peaked on day 14 (Fig. 7). In this group, γδ T cells were the T cell subset with the highest proportion of cells producing IFN-γ, TNF or IL-2. When combining data for all individuals in group B at each time point, and comparing responses to day 0, CD8^+^ T cells producing IFN-γ were significantly increased at day 14 (*p* = 0.007) and CD4^+^ T cells and γδ T cells secreting TNF were also significantly increased at day 14 (*p* = 0.040 and 0.036 respectively).

## Discussion

Here, for the first time, we describe the preparation and administration to humans of chemically treated Pf pRBC and show that a single dose of attenuated Pf pRBC is able to induce strain and species-transcending cellular immune responses in malaria-naïve individuals. Pf-specific antibody responses were not detected. The dose-ranging component of this study indicates that doses of > 50 nM TF-A must be used in vitro to effectively attenuate *P. falciparum* and prevent the development of the clinical manifestations of Pf infection in vivo.

Lymphoproliferative responses were examined, and as previously shown, all individuals had pre-existing responses to pRBC at baseline [36], despite no previous exposure to Pf. This responsiveness could be due to cross-reactivity between parasite antigens and environmental organisms [36]. Critically, the responses to homologous pRBC increased following inoculation in individuals who received completely attenuated chemically treated Pf 7G8 (group B), and they persisted in a proportion of individuals for up to 90 days (the duration of the study). Proliferative responses to heterologous parasites were also observed in more than half of these individuals. It is possible that administration of additional doses of attenuated parasites will augment the breadth, magnitude and persistence of this response. Cytokine production in response to homologous parasites was also examined, and increased production of IFN-γ and TNF (as measured in culture supernatants) was observed following inoculation. Both IFN-γ and TNF are strongly implicated in protection against Pf infection [25–28, 37], and the latter also in pathology [38–40]. Increased levels of IL-10 were also observed; it is a regulatory cytokine thought to play a crucial role in *Plasmodium* spp. infection, due to its ability to regulate both innate and adaptive inflammatory responses, e.g. production of TNF (reviewed in [41]).

By using intracellular cytokine staining, we further examined production of IFN-γ, TNF and IL-2 by CD3^+^ T cells. In group B, T cells secreting IFN-γ alone or in combination with TNF were the most frequently detected; however, a significant increase following vaccination was only seen at day 14 for CD3^+^ T cells secreting IFN-γ alone. Previous studies on malaria and other infectious diseases have demonstrated a correlation between the presence of antigen-specific polyfunctional T cells and protective immunity induced following vaccination (e.g. [42–44]). Although the prevalence of these cells was low and the increases in these populations following vaccination were not significant, administration of additional vaccine doses may increase their prevalence and longevity. Future studies examining the protective efficacy of a chemically attenuated whole blood-stage parasite vaccine should examine the role of these polyfunctional T cells in protection. Importantly, we also observed the induction of cytokine-secreting memory T cells (CD3^+^CD45RO^+^) following inoculation. It would be of interest in future studies to undertake additional phenotyping to examine the pluripotency of this memory T cell population. Analysis of the CD3^+^ T cell subsets following inoculation showed that there was a significant increase at day 14 in CD4^+^ and γδ T cells producing TNF and in CD8^+^ T cells producing IFN-γ.

Pf-specific IgM was detected only in individuals who developed an active malaria infection (group A). The lack of antibody production in group B may be a function of antigen dose, as they were exposed to a lower dose of parasites. It is possible that with administration of further doses of the completely attenuated parasites, an antibody response may be induced.

In a previous clinical study investigating the protective efficacy of multiple low-dose Pf infections attenuated in vivo with atovaquone/proguanil, we observed protection against homologous challenge in three out of four volunteers [29]. However, we could not exclude that protection was due in part to residual drug [45]. Pf-specific cellular immune responses were induced in the absence of Pf-specific antibody in that study, similarly to what we observed in the current study. However, the requirement for delayed anti-malarial drug administration is problematic for the feasibility of this in vivo treatment approach as a vaccine strategy. Our current approach of in vitro treatment of pRBC prior to administration offers a viable alternative.

Induction of parasite-specific cellular immune responses, in the absence of antibodies, was observed in previous rodent studies evaluating the protective efficacy of chemically attenuated *P. chabaudi* pRBC [18]. This is similar to what we observed in the current study, and it differs from the *P. yoelii* 17X rodent studies, where parasite-specific antibodies were also induced in addition to the cellular immune responses [19]. A further study involving administration of chemically treated Pf FVO parasites to non-splenectomised *Aotus* monkeys demonstrated induction of Pf-specific cellular responses in the absence of IgG [30]. Following a single dose of chemically treated parasites, the vaccinated monkeys received a homologous blood-stage challenge with all developing parasitemia and requiring anti-malarial drug treatment. This suggests that more than one vaccine dose may be required for clinical protection.

Rodent studies investigating chemically attenuated *Plasmodium* spp. suggest that persistence of low levels of parasite antigen may be important for inducing an antibody-independent protective immune response [20]. Although persisting parasites could not be detected in this current study beyond day 2 post-inoculation, it is possible that they were persisting at levels below the limit of detection of the qPCR.

The development and clinical evaluation of this whole parasite asexual blood-stage vaccine approach presented a number of general and specific issues for consideration. The use of human red blood cells in the manufacturing process and the final vaccine product entailed specific regulatory and safety considerations, specifically the possibility of contamination with infectious adventitious agents and allo-immunisation. To address the first issue, we used transfusion-compatible blood products, with collection and screening undertaken according to current regulatory guidelines, and used a defined malaria parasite cell bank grown at Good Manufacturing Practices (GMP) standard that had also been rigorously screened according to regulatory guidelines [31]. Furthermore, the manufacturing process complied with current, local GMP requirements. The second issue, the possibility of allo-immunisation (the induction of antibodies against red blood cell antigens), was addressed by the use of blood from a group O RhD negative donor to manufacture the inoculum for group A and the first three participants in group B (P4-P6). In one study participant, P6, seroconversion to the minor Rh antigen “c” was observed following inoculation. As the Rh phenotype of the donor red blood cells that were used to manufacture the inoculum for the first three participants in group B were “ce” while P6’s phenotype was “CDe”, it is likely that the induction of “c” antibodies may have been due to injection with the chemically treated pRBC. Seroconversion was not observed in P1–P5 despite incompatibility with the donor red blood cells at the minor Rh antigens. Although it is not feasible to match donor blood with recipients at all of the minor Rh antigens, following this observation, the inocula for P7 and P8 were manufactured individually using their own red blood cells. In this current study, the inocula were prepared from cultures with a 5% parasitemia; thus, the total number of red blood cells being injected was 20-fold higher than the number of pRBC. To progress this vaccine strategy and to address the possible induction of alloantibodies, we believe it is critical to reduce the number of red blood cells in the inocula, which could be achieved by purifying the pRBC away from the uninfected red blood cells. This current study involved the administration of only a single dose of Pf pRBC; the impact of multiple doses of Pf pRBC on the induction of alloantibodies is being examined in ongoing studies.

TF-A is a compound with genotoxic potential, and while the majority is washed away during the manufacturing process, its use required the measurement of residual TF-A in an inoculum dose for each manufactured batch. According to the “EU Guidelines on the limits of Genotoxic Impurities” (which has been adopted by our local regulatory body, the Therapeutic Goods Administration), a value of 1.5 μg/day of genotoxic impurity is considered to be associated with acceptable risk. Notably, the US FDA stipulates a much higher threshold of 120 μg/day, and this is for up to 14 days of continuous administration. The amount of residual TF-A in our inoculum batches was considerably lower than both of these thresholds (group A: *x* = 86.04 ng/vaccine dose; group B: *x* = 114 ng/vaccine dose). Purification of pRBC away from uRBC would result in an even further reduction in the amount of residual TF-A.

## Conclusions

This study represents the first clinical evaluation of chemically attenuated whole blood-stage parasites in malaria-naïve human volunteers. When the Pf parasites were completely attenuated, the inoculum was safe and well tolerated, although future studies may need to focus on the purification of pRBC (e.g. magnet purification of trophozoite-stage pRBC) for the inoculum to address the possibility of induction of alloantibodies. The induction of strain and species-transcending parasite-specific cellular immune responses following inoculation provides support for the whole blood-stage parasite approach as a means of increasing the breadth of the resulting immune response. While homologous and heterologous protection has been demonstrated in rodent models of malaria [18, 19], it is not known whether these cross-reactive immune responses will be protective in humans. These data support further clinical development of chemically attenuated whole blood-stage parasites as a vaccine strategy. Future studies will focus on a multi-dose immunisation regimen and will address whether lower doses of attenuated parasites are immunogenic.

## Additional file

Additional file 1: **Table S1.** Demographics of study participants. **Table S2.** Adverse events reported in study participants in group A. **Table S3.** Abnormal laboratory values reported in study participants in group A. **Figure S1.** In vitro growth of *P. falciparum* 7G8 following treatment with different doses of tafuramycin-A. **Figure S2.** Induction of *P. falciparum* 7G8 IgG responses in study participants inoculated with a single dose of (A) 3 × 10^7^*P. falciparum* 7G8 pRBC treated with 50 nM of TF-A or (B) 3 × 10^7^*P. falciparum* 7G8 pRBC treated with 200 nM of TF-A. **Figure S3.** Cytokine responses to *P. falciparum* 7G8 in study participants inoculated with a single dose of (A) 3 × 10^7^*P. falciparum* 7G8 pRBC treated with 50 nM (group A) or (B) 200 nM (group B) TF-A. **Figure S4.** Serum cytokine responses in study participants inoculated with a single dose of (A) 3 × 10^7^*P. falciparum* 7G8 pRBC treated with 50 nM TF-A (group A) or (B) 1,800 *P. falciparum* 7G8 pRBC (infectivity study) untreated. **Figure S5.** Monofunctional and polyfunctional CD3^+^ T cells in study participants inoculated with a single dose of 3 × 10^7^*P. falciparum* 7G8 pRBC treated with 50 nM TF-A (group A). **Figure S6.** Cytokine production in naïve and memory T lymphocytes in study participants inoculated with a single dose of 3 × 10^7^*P. falciparum* 7G8 pRBC treated with 50 nM TF-A (group A). **Figure S7.** Cytokine production in CD3^+^ lymphocyte sub-populations in study participants inoculated with a single dose of 3 × 10^7^*P. falciparum* 7G8 pRBC treated with 50 nM TF-A (group A). (PDF 675 kb)

## Abbreviations

A/L: Artemether-lumefantrine
BSA: Bovine serum albumin
CBA: Cytometric bead array
CCPM: Corrected counts per minute
CIPDD: Centre for Integrated Preclinical Drug Development
CM: Centanamycin
CPM: Counts per minute
ELISA: Enzyme-linked immunosorbent assay
EU: European Union
FBS: Foetal bovine serum
GMP: Good Manufacturing Practices
IFN: Interferon
IgG: Immunoglobulin G
IgM: Immunoglobulin M
IL: Interleukin
IU: International units
ng: Nanogram
nM: Nanomolar
PBMC: Peripheral blood mononuclear cells
PBS: Phosphate buffered saline
Pf: *Plasmodium falciparum*
PHA: Phytohaemagglutinin
PIC/S: Pharmaceutical Inspection Co-operation Scheme
pRBC: Parasitized red blood cells
qPCR: Quantitative polymerase chain reaction
RPMI: Roswell Park Memorial Institute medium
TF-A: Tafuramycin A
TMB: Tetramethylbenzadine
TNF: Tumour necrosis factor
U: Units
uRBC: Uninfected red blood cells
