# Supplementary material for: Vaccination with chemically attenuated Plasmodium falciparum asexual blood-stage parasites induces parasite-specific cellular immune responses in malaria-naïve volunteers: a pilot study
Source: BMC Med. 2018 Oct 8;16:184. doi: 10.1186/s12916-018-1173-9 (PMC6174572; doi:10.1186/s12916-018-1173-9)
Supplement: Supplementary file 1 — Table S1. Demographics of study participants. Table S2. Adverse events reported in study participants in group A. Table S3. Abnormal laboratory values reported in study participants in group A. Figure S1. In vitro growth of P. falciparum 7G8 following treatment with different doses of tafuramycin-A. Figure S2. Induction of P. falciparum 7G8 IgG responses in study participants inoculated with a single dose of (A) 3 × 10^7^P. falciparum 7G8 pRBC treated with 50 nM of TF-A or (B) 3 × 10^7^P. falciparum 7G8 pRBC treated with 200 nM of TF-A. Figure S3. Cytokine responses to P. falciparum 7G8 in study participants inoculated with a single dose of (A) 3 × 10^7^P. falciparum 7G8 pRBC treated with 50 nM (group A) or (B) 200 nM (group B) TF-A. Figure S4. Serum cytokine responses in study participants inoculated with a single dose of (A) 3 × 10^7^P. falciparum 7G8 pRBC treated with 50 nM TF-A (group A) or (B) 1,800 P. falciparum 7G8 pRBC (infectivity study) untreated. Figure S5. Monofunctional and polyfunctional CD3+ T cells in study participants inoculated with a single dose of 3 × 10^7^P. falciparum 7G8 pRBC treated with 50 nM TF-A (group A). Figure S6. Cytokine production in naïve and memory T lymphocytes in study participants inoculated with a single dose of 3 × 10^7^P. falciparum 7G8 pRBC treated with 50 nM TF-A (group A). Figure S7. Cytokine production in CD3+ lymphocyte sub-populations in study participants inoculated with a single dose of 3 × 10^7^P. falciparum 7G8 pRBC treated with 50 nM TF-A (group A). (PDF 675 kb) [file 12916_2018_1173_MOESM1_ESM.pdf]

Table S1: Demographics of study participants

	**Group A**	**Group B**
Subjects enrolled, n	3	5
Age at enrolment (yrs); mean (SD)	35 (20.9)	35.6 (15.5)
Age Range (min, max)	21, 59	24, 54
Male, n (%)	3 (100)	5 (100)
Race, n (%)		
Caucasian	3 (100)	5 (100)
BMI (kg/m2)		
Mean (SD)	26.1 (1.7)	24.2 (1.2)
Range (min, max)	25.1, 28	22.2, 25.2

Table S2: Adverse events reported in study participants in Group A[a]

Symptom	Severity-Mild	Severity-Moderate	Severity-Severe	Total
Malaise	2[c,c]	0	0	2
Headache	2[c,f]	1[c]	0	3
Myalgia	1[c]	0	0	1
Fever (objective or subjective)	1[d]	2[c,c]	1[d]	4
Tachycardia	1[c]	0	0	1
Sweating	2[c,d]	0	0	2
Rigors	0	2[d,d]	0	2
Pain in metacarpal	1[e]	0	0	1
Pain right ischial tuberosity	1[b]	0	0	1
Aching sensation in knees, elbows, shoulders	0	1[c]	0	1
Abdominal pain	1[c]	0	0	1
Frequent loose stools	1[c]	0	0	1
Neck and back pain	1[c]	0	0	1
Chills	1[d]	0	0	1

[a]Only those adverse events deemed possibly or probably related to the investigational product (ie chemically attenuated pRBC) are reported.

[b]Occurred in the 8 day period post injection of the investigational product

[c]Occurred on the day prior to/day of initiation of anti-malarial treatment, but prior to treatment commencing

[d]Occurred in the anti-malarial treatment period

[e]Occurred in the time period following completion of anti-malarial treatment

[f]Occurred on day 9 post injection; initiation of anti-malarial treatment in this participant was on day 11.

Table S3: Abnormal laboratory values reported in study participants in Group A[a]

Symptom	Severity-Mild	Severity-Moderate	Severity-Severe	Total
Elevated AST	1[c]	0	0	1
Lymphocytopenia	1[d]	1[c]	0	2
Thrombocytopenia	2[d,d]	0	1[d]	3
Elevated atypical lymphocytes	1[d]	1[d]	0	2
Low red blood cell count	1[d]	0	0	1
High monocytes	1[d]	0	0	1
Neutropenia	2[b,b]	0	1[d]	3
Elevated eosinophils	1[b]	0	0	1
Leukopenia	0	0	1[c]	1

[a]Only those abnormal laboratory values deemed possibly or probably related to the investigational product (ie chemically attenuated pRBC) are reported.

[b]Occurred in the 8 day period post injection of the investigational product

[c]Occurred on the day prior to/day of initiation of anti-malarial treatment, but prior to treatment commencing

[d]Occurred in the anti-malarial treatment period

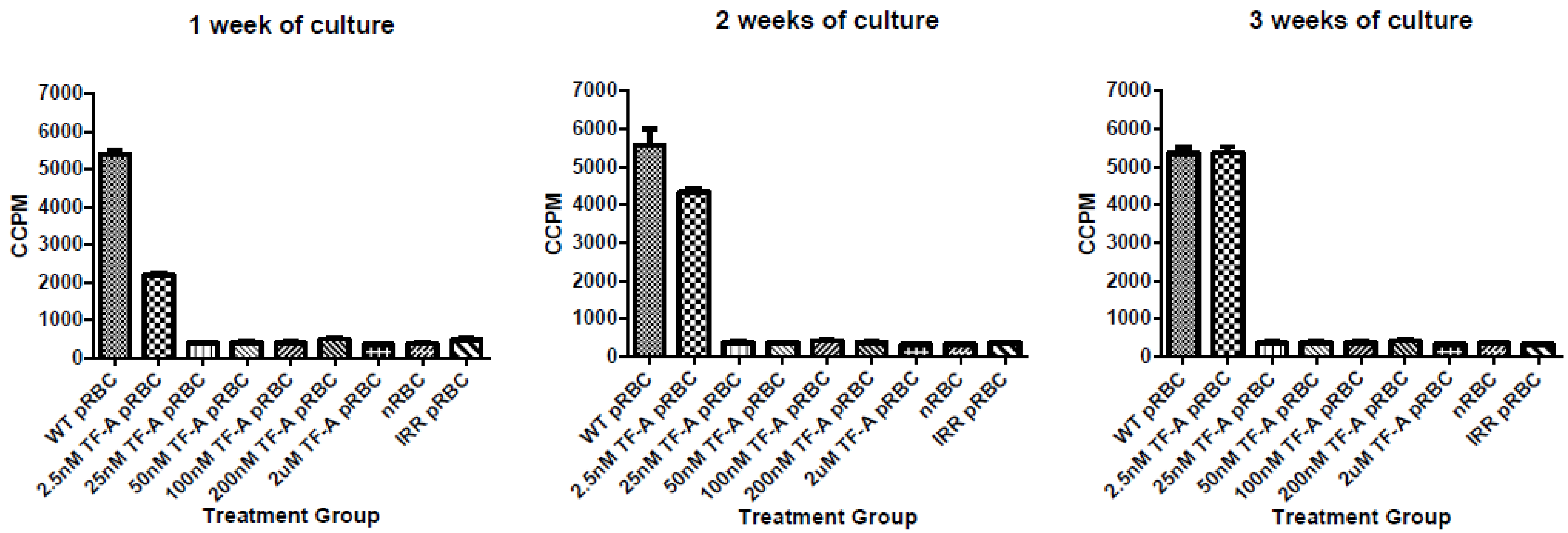

Figure S1: *In vitro* growth of *P. falciparum* 7G8 following treatment with different doses of Tafuramycin-A. Parasite growth was evaluated by ^{3}[H] hypoxanthine incorporation. Untreated wild-type parasitised red blood cells (WT pRBC), parasitised red blood cells (pRBC) treated with different doses of TF-A (2.5nM-2µM), normal red blood cells (nRBC) and irradiated pRBC (IRR pRBC) were cultured for one, two or three weeks. At the end of each week, pRBC from each treatment group were harvested from continuous cultures and incubated with ^{3}[H] hypoxanthine for 48 hours. Data represents mean ± SEM for each time point (n=5 wells/treatment/time point). CCPM: counts per minutes.

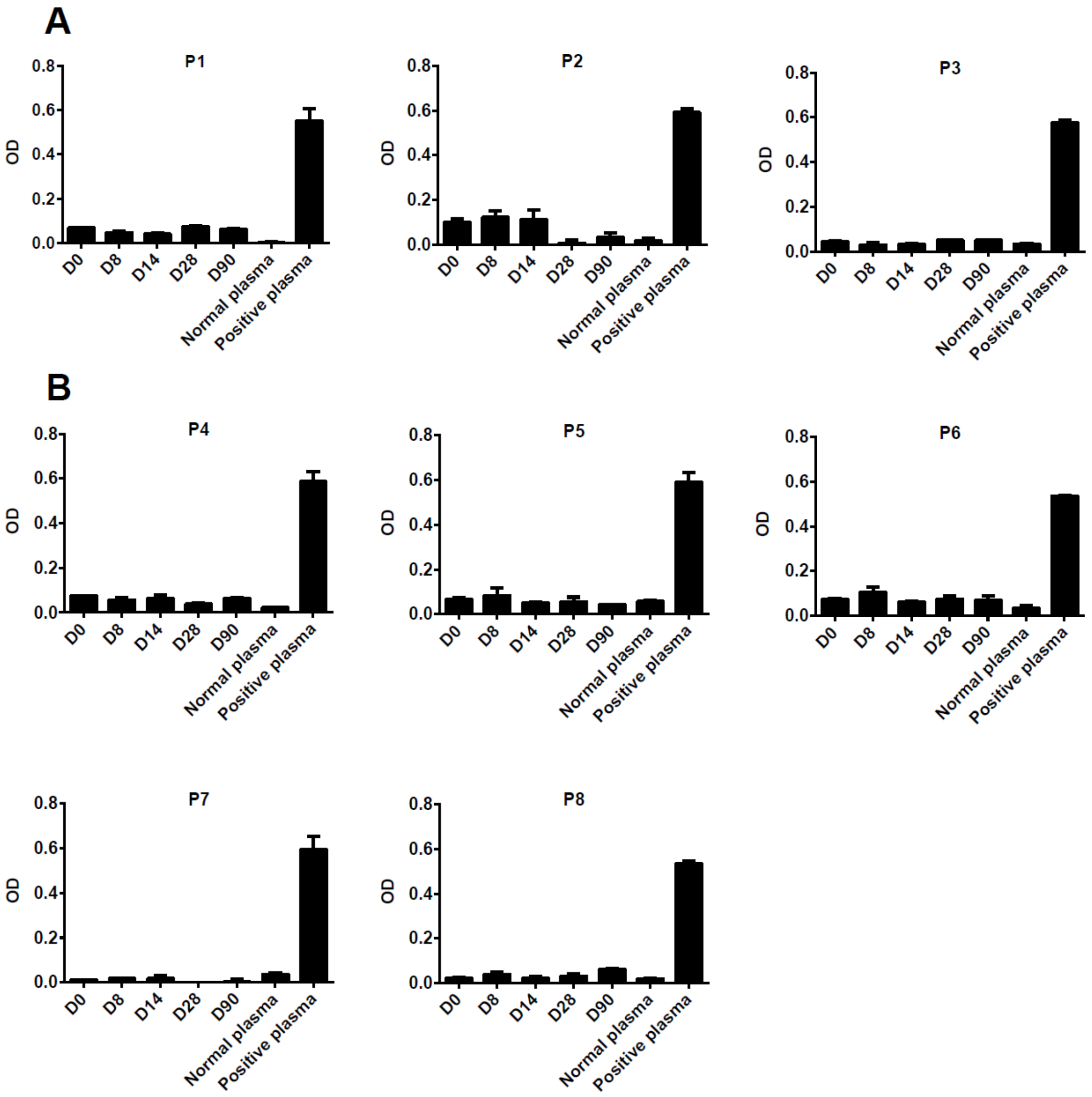

Figure S2: Induction of *P. falciparum* 7G8 IgG responses in study participants inoculated with a single dose of (A) 3 x 10^7 *P. falciparum* 7G8 pRBC treated with 50nM of TF-A (B) 3 x 10^7 *P. falciparum* 7G8 pRBC treated with 200nM of TF-A. ELISAs were performed to detect IgG specific for crude *P. falciparum* 7G8 antigen using plasma collected at different time points post vaccination. Results are expressed as optical density (OD) at 650nm. Samples were run in duplicate. Data represents mean ± SEM. An individual's data were analysed using a one-way ANOVA followed by Dunnett's multiple comparisons test.

IgG responses post inoculation were not significantly different from baseline at any time point for any individual ($p>0.05$).

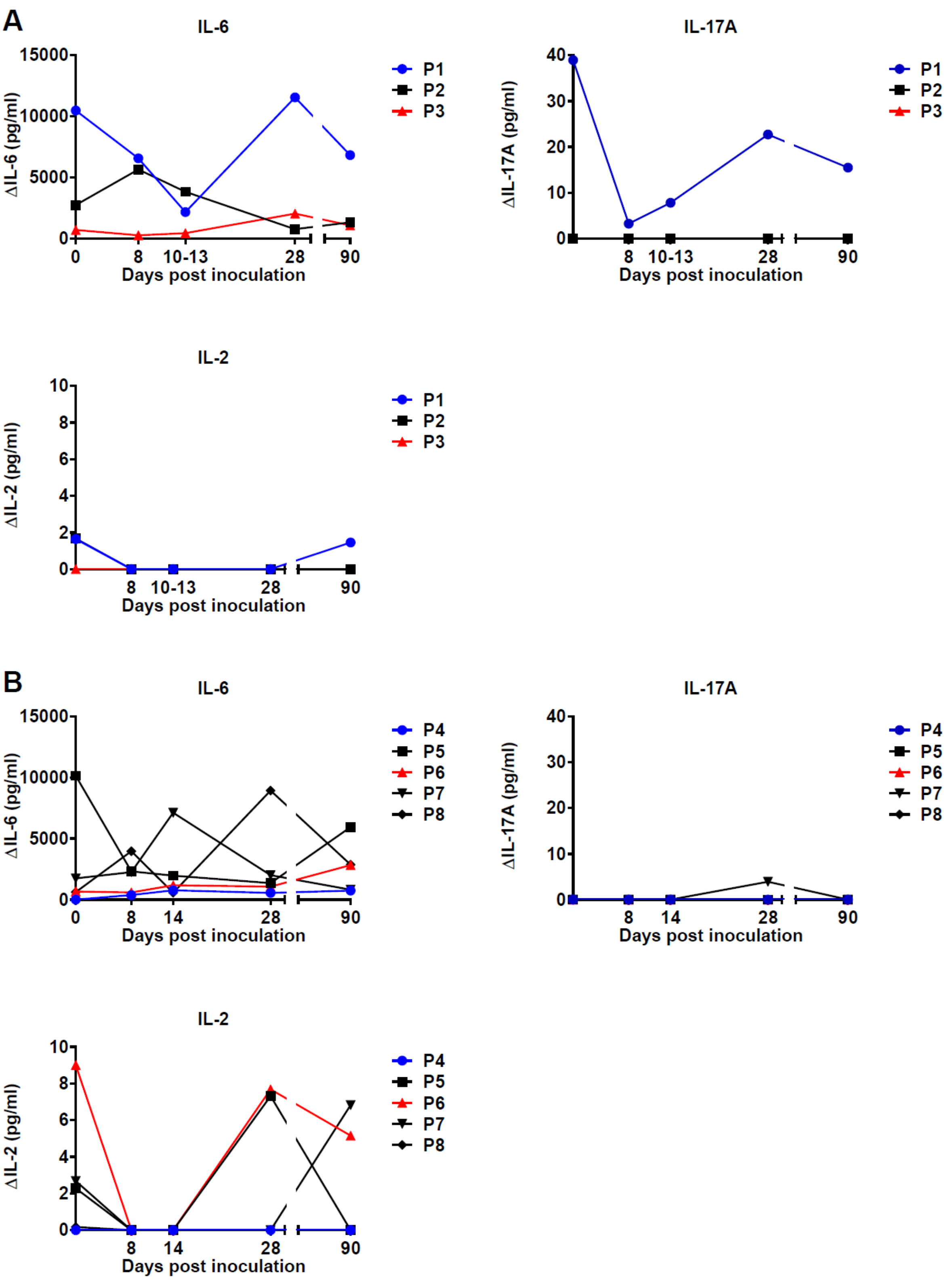

Figure S3: Cytokine responses to *P. falciparum* 7G8 in study participants inoculated with a single dose of (A) 3 x 10^7 *P. falciparum* 7G8 pRBC treated with 50nM (Group A) or (B) 200nM (Group B) TF-A. Peripheral blood mononuclear cells (PBMCs) were isolated

from blood samples collected at different time points post inoculation and cryopreserved. Following thawing, PBMCs were incubated with parasitised red blood cells (pRBC) or unparasitised red blood cells (uRBC) for 7 days. Eighteen hours before the end of the culture period, culture supernatants were collected, pooled (n=3) and used in cytokine bead arrays to quantify the level of cytokines produced in response to *P. falciparum 7G8* pRBCs by flow cytometric analysis. Delta cytokine indicates that responses to pRBC were corrected against responses to uRBC.

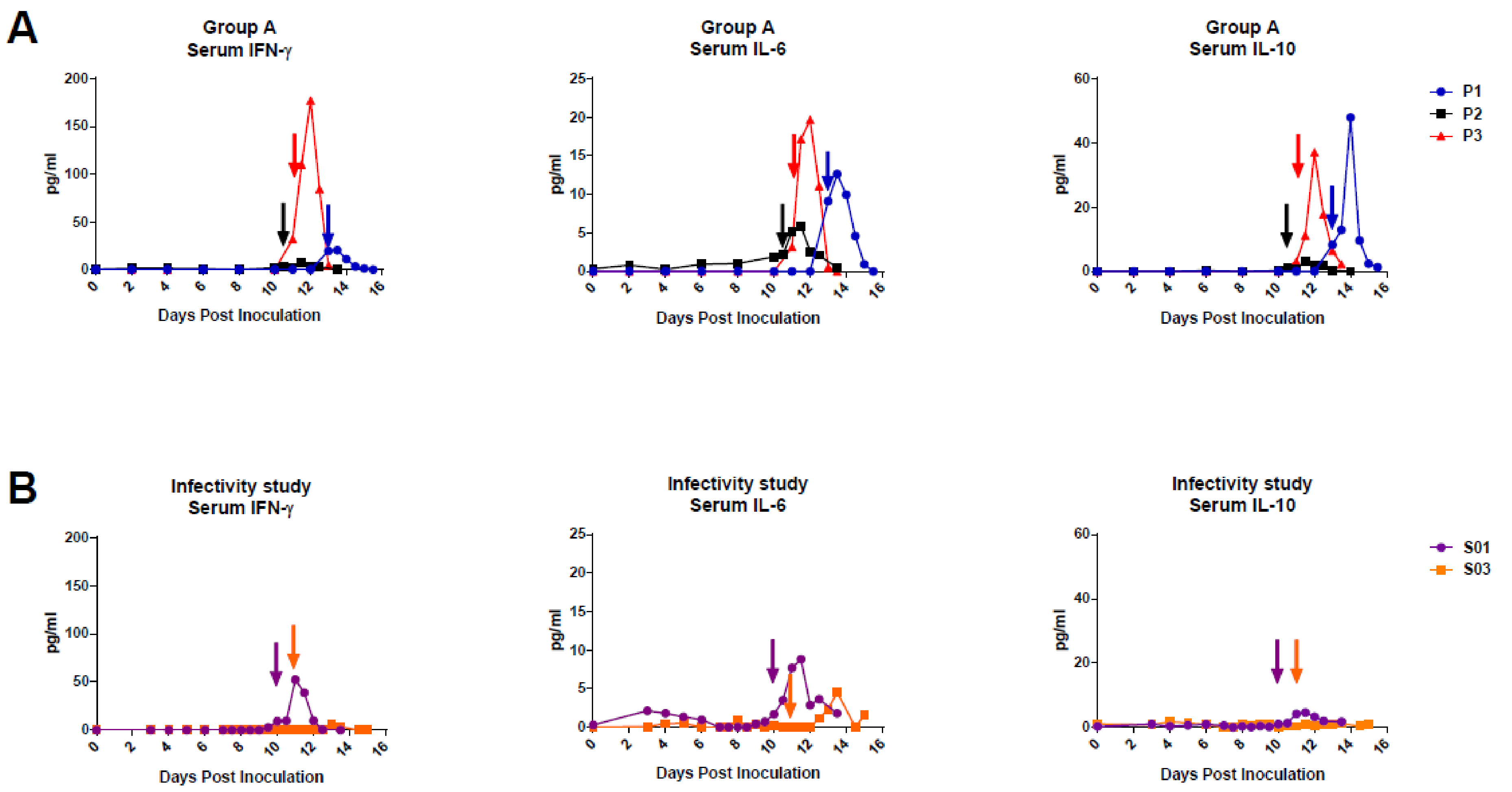

Figure S4: Serum cytokine responses in study participants inoculated with a single dose of (A) 3 x 10^7 *P. falciparum* 7G8 pRBC treated with 50nM TF-A (Group A) or (B) 1,800 *P. falciparum* 7G8 pRBC (Infectivity study) untreated. Serum was processed from blood samples

collected at different points post inoculation and frozen. Samples that were collected from Group A in the current study or from a previous study where we had evaluated the infectivity of the Pf 7G8 cell bank (Stanisic et al *Infect Immun.* 2016;84(9):2689-96) were used in cytokine bead arrays to quantify the level of cytokines produced by flow cytometric analysis. Arrows indicate initiation of drug treatment with Artemether-Lumefantrine.

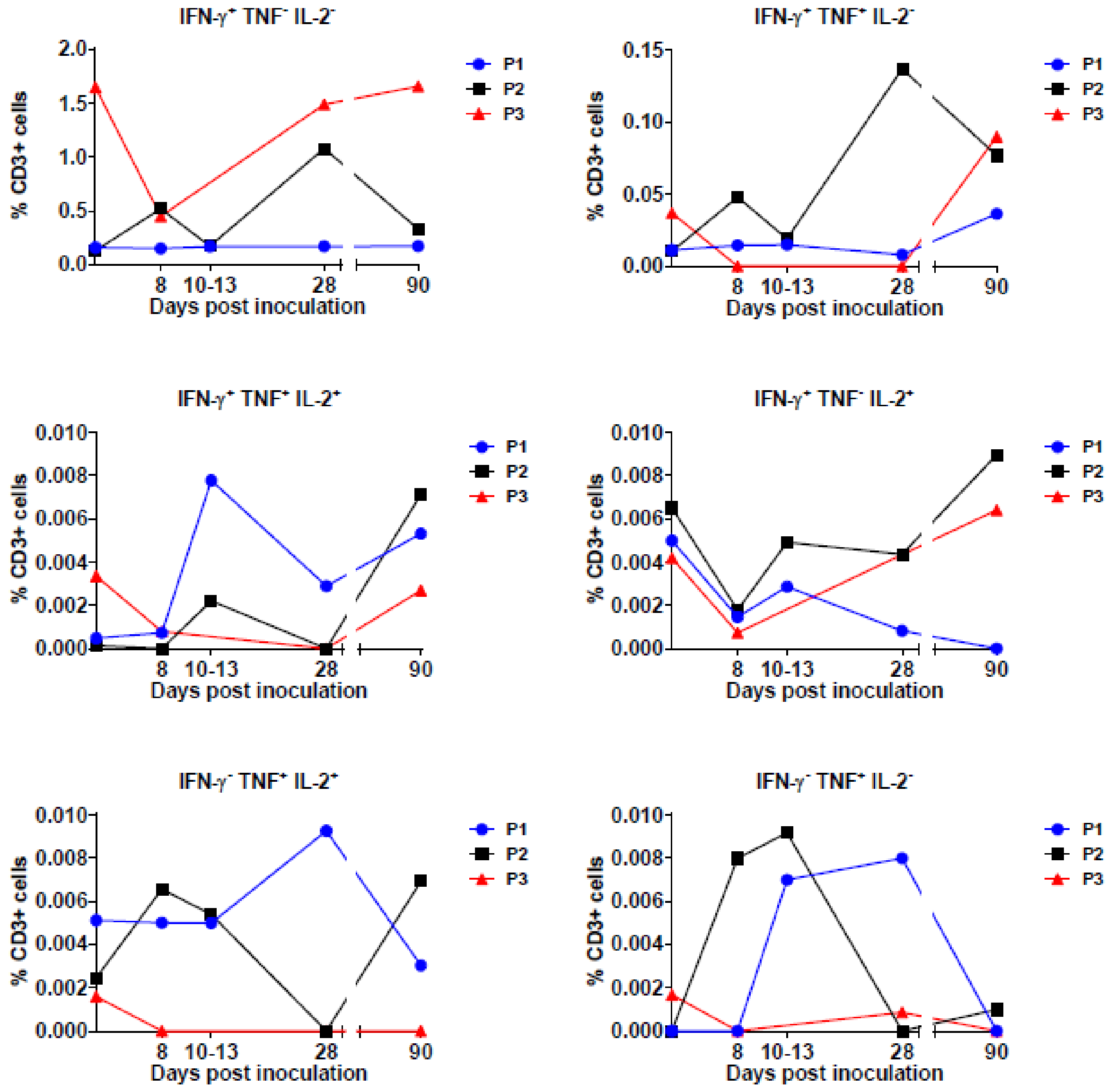

Figure S5: Monofunctional and polyfunctional CD3$^+$ T cells in study participants inoculated with a single dose of 3 x 10^7 *P. falciparum* 7G8 pRBC treated with 50nM TF-A (Group A). Peripheral blood mononuclear cells (PBMCs) were isolated from blood samples collected at different time points post inoculation and cryopreserved. Following thawing, PBMCs were incubated with parasitised red blood cells (pRBC) or unparasitised red blood cells (uRBC) for 36 hours. Cells from triplicate wells were collected and pooled prior to staining with antibodies for flow cytometric analysis to evaluate the proportion of CD3$^+$ T

cells producing intracellular IFN-γ, TNF and IL-2. Responses to pRBC were corrected against responses to uRBC. The Day 10-13 sample for P3 was not available for testing.

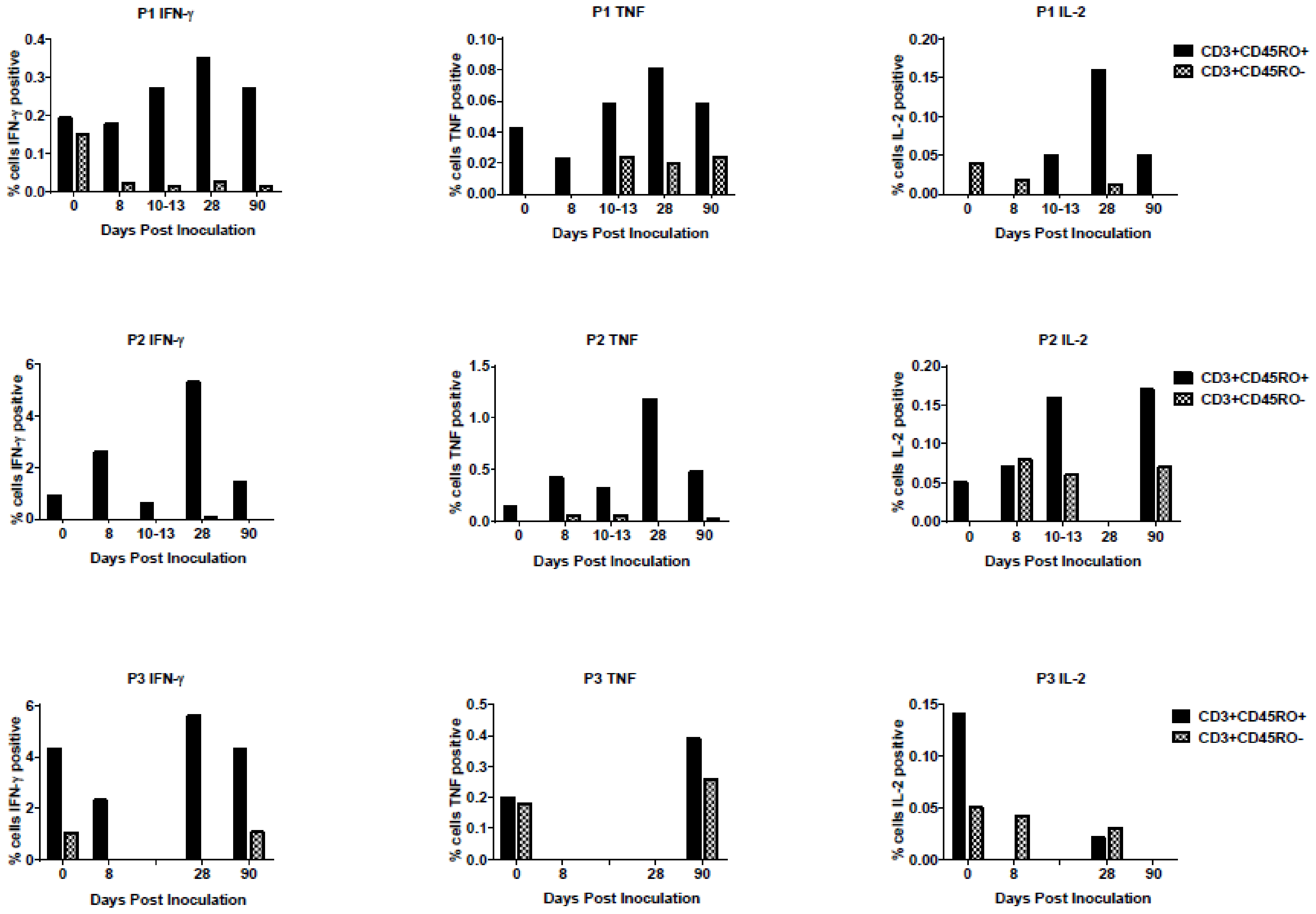
P1 IFN-γ
% cells IFN-γ positive
0.0
0.1
0.2
0.3
0.4
0
8
10-13
28
90
Days Post Inoculation
P1 TNF
% cells TNF positive
0.00
0.02
0.04
0.06
0.08
0.10
0
8
10-13
28
90
Days Post Inoculation
P1 IL-2
% cells IL-2 positive
0.00
0.05
0.10
0.15
0.20
0
8
10-13
28
90
Days Post Inoculation
CD3+CD45RO+
CD3+CD45RO-
P2 IFN-γ
% cells IFN-γ positive
0
2
4
6
0
8
10-13
28
90
Days Post Inoculation
P2 TNF
% cells TNF positive
0.0
0.5
1.0
1.5
0
8
10-13
28
90
Days Post Inoculation
P2 IL-2
% cells IL-2 positive
0.00
0.05
0.10
0.15
0.20
0
8
10-13
28
90
Days Post Inoculation
CD3+CD45RO+
CD3+CD45RO-
P3 IFN-γ
% cells IFN-γ positive
0
2
4
6
0
8
28
90
Days Post Inoculation
P3 TNF
% cells TNF positive
0.0
0.1
0.2
0.3
0.4
0.5
0
8
28
90
Days Post Inoculation
P3 IL-2
% cells IL-2 positive
0.00
0.05
0.10
0.15
0
8
28
90
Days Post Inoculation
CD3+CD45RO+
CD3+CD45RO-

Figure S6: Cytokine production in naïve and memory T lymphocytes in study participants inoculated with a single dose of 3 x 10^7 *P. falciparum* 7G8 pRBC treated with 50nM TF-A (Group A). Peripheral blood mononuclear cells (PBMCs) were isolated from blood samples collected at different time points post inoculation and cryopreserved. Following thawing, PBMCs were incubated with parasitised red blood cells (pRBC) or unparasitised red blood cells (uRBC) for 36 hours. Cells from triplicate wells were collected and pooled prior to staining with antibodies for flow cytometric analysis to evaluate the proportion of naïve T cells ($CD3^+CD45RO^-$), and memory T cells ($CD3^+CD45RO^+$) producing intracellular IFN-γ, TNF and IL-2. Responses to pRBC were against responses to uRBC. The Day 10-13 sample for P3 was not available for testing.

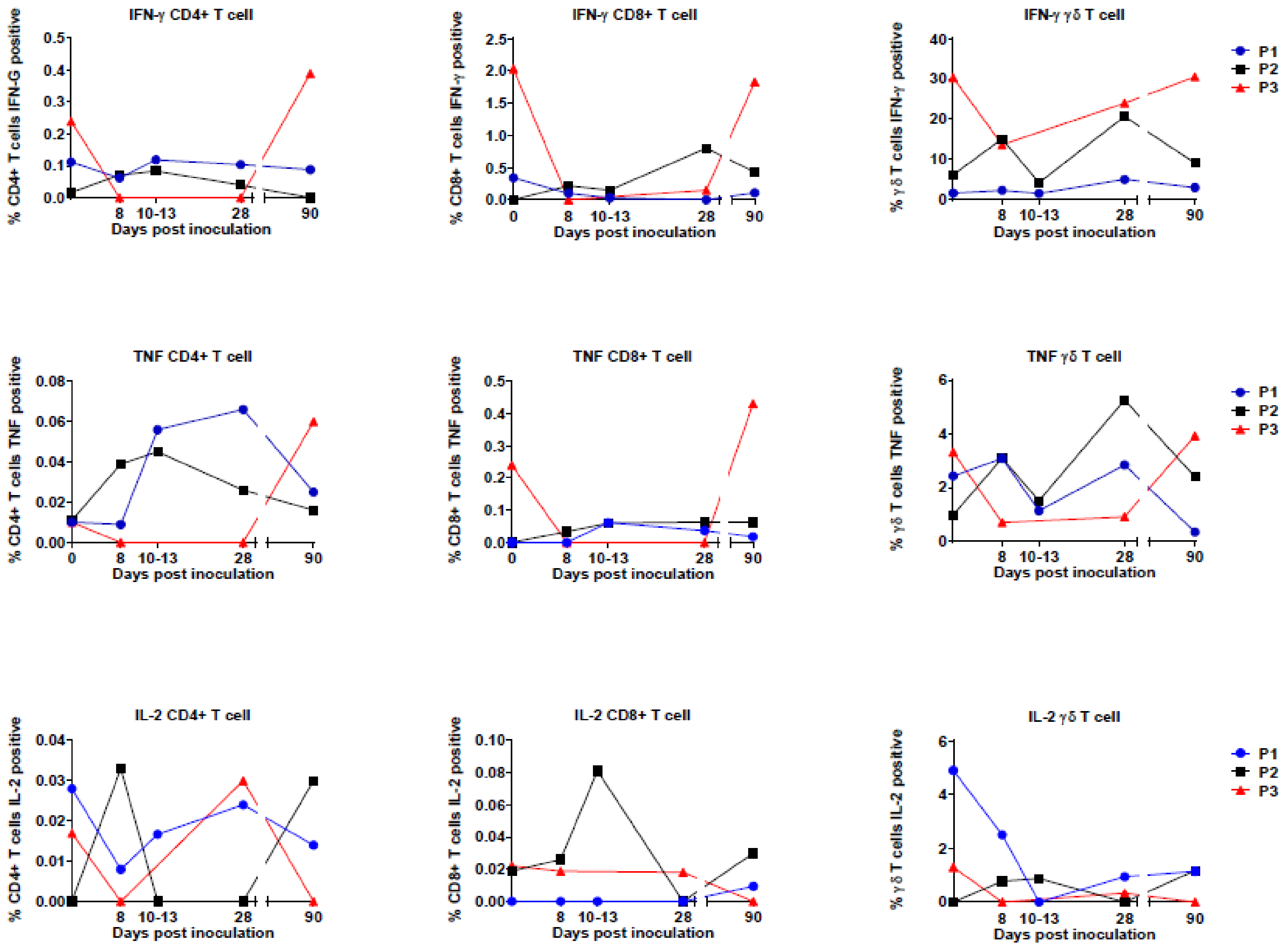
IFN-γ CD4+ T cell
% CD4+ T cells IFN-G positive
0.0
0.1
0.2
0.3
0.4
0.5
8
10-13
28
90
Days post inoculation
IFN-γ CD8+ T cell
% CD8+ T cells IFN-γ positive
0.0
0.5
1.0
1.5
2.0
2.5
0
8
10-13
28
90
Days post inoculation
IFN-γ γδ T cell
% γδ T cells IFN-γ positive
0
10
20
30
40
8
10-13
28
90
Days post inoculation
P1
P2
P3
TNF CD4+ T cell
% CD4+ T cells TNF positive
0.00
0.02
0.04
0.06
0.08
0
8
10-13
28
90
Days post inoculation
TNF CD8+ T cell
% CD8+ T cells TNF positive
0.0
0.1
0.2
0.3
0.4
0.5
0
8
10-13
28
90
Days post inoculation
TNF γδ T cell
% γδ T cells TNF positive
0
2
4
6
8
10-13
28
90
Days post inoculation
P1
P2
P3
IL-2 CD4+ T cell
% CD4+ T cells IL-2 positive
0.00
0.01
0.02
0.03
0.04
8
10-13
28
90
Days post inoculation
IL-2 CD8+ T cell
% CD8+ T cells IL-2 positive
0.00
0.02
0.04
0.06
0.08
0.10
8
10-13
28
90
Days post inoculation
IL-2 γδ T cell
% γδ T cells IL-2 positive
0
2
4
6
8
10-13
28
90
Days post inoculation
P1
P2
P3

Figure S7: Cytokine production in $CD3^+$ lymphocyte sub-populations in study participants inoculated with a single dose of 3 x 10^7 *P. falciparum* 7G8 pRBC treated with 50nM TF-A (Group A). Peripheral blood mononuclear cells (PBMCs) were isolated from blood samples collected at different time points post inoculation and cryopreserved. Following thawing, PBMCs were incubated with parasitised red blood cells (pRBC) or unparasitised red blood cells (uRBC) for 36 hours. Cells from triplicate wells were collected and pooled prior to staining with antibodies for flow cytometric analysis to evaluate intracellular IFN-γ, TNF and IL-2 production in in helper T cells ($CD3^+CD4^+CD8^-$), cytotoxic T cells ($CD3^+CD4^-CD8^+$) and γδ T cells ($CD3^+$ $\gamma\delta^+$). Responses to pRBC were against responses to nRBC. The Day 10-13 sample for P3 was not available for testing.
